# The Significance of Discordant Serology in Chagas Disease: Enhanced T-Cell Immunity to *Trypanosoma cruzi* in Serodiscordant Subjects

**DOI:** 10.3389/fimmu.2017.01141

**Published:** 2017-09-15

**Authors:** Melisa D. Castro Eiro, María G. Alvarez, Gretchen Cooley, Rodolfo J. Viotti, Graciela L. Bertocchi, Bruno Lococo, María C. Albareda, Ana M. De Rissio, María A. Natale, Cecilia Parodi, Rick L. Tarleton, Susana A. Laucella

**Affiliations:** ^1^Instituto Nacional de Parasitología ‘Dr. Mario Fatala Chabén’, Buenos Aires, Argentina; ^2^Hospital Interzonal General de Agudos Eva Perón, Buenos Aires, Argentina; ^3^Center for Tropical and Emerging Global Diseases, University of Georgia, Athens, GA, United States; ^4^Instituto de Patología Experimental (CONICET), Universidad Nacional de Salta, Salta, Argentina

**Keywords:** *Trypanosoma cruzi*, serodiscordance, T cells, multiplex assay, polyfunctionality

## Abstract

**Background:**

Subjects are considered infected with *Trypanosoma cruzi* when tested positive by at least two out of three serological tests, whereas a positive result in only one of up to three tests is termed “serodiscordant” (SD). Assessment of parasite-specific T-cell responses may help discriminate the uninfected from infected individuals among SD subjects.

**Methods:**

Peripheral blood mononuclear cells from SD and seropositive (SP) subjects, who were born in areas endemic for *T. cruzi* infection but living in Buenos Aires city, Argentina, at the time of the study, and seronegative unexposed subjects were included for analysis. The function and phenotype of T cells were assessed by interferon-γ (IFN-γ) and interleukin (IL)-2 enzyme-linked immunospot assay and multiparameter flow cytometry. *T. cruzi*-specific antibodies were quantified by conventional serology and a multiplex assay format.

**Results:**

SD subjects exhibited immunity cell responses to *T. cruzi* but in contrast to SP subjects, T cells in SD subjects more often display the simultaneous production of IFN-γ and IL-2 in response to *T. cruzi* antigens and have a resting phenotype. SD individuals also have higher IFN-γ spot counts, polyfunctional CD4^+^ T-cells enriched in IL-2 secreting cells and low levels of antibodies specific for a set of *T. cruzi*-derived recombinant proteins compared with the SP group. Long-term follow-up of SD individuals confirmed that humoral and T-cell responses fluctuate but are sustained over time in these subjects. T cells in SD subjects for *T. cruzi* infection did not recognize *Leishmania* antigens.

**Conclusion:**

Both T-cell and humoral responses in most subjects assessed by conventional tests as SD for *T. cruzi* infection indicate prior exposure to infection and the establishment of immunological memory suggestive of a resolved infection.

## Introduction

Chagas disease, caused by the intracellular pathogen *Trypanosoma cruzi*, is the highest impact parasitic disease in Latin America and the most common cause of infectious myocarditis in the world (1). T-cell function, including the production of cytokines, proliferative capacity and the ability to exert cytotoxicity are central to the control of intracellular pathogens (2–4). The role of CD4^+^ and CD8^+^ T lymphocytes in the control of *T. cruzi* infection has been demonstrated by studies showing the predominance of these cells in inflammatory infiltrates of parasitized tissues (5–9) and the increased parasite burden in tissues of mice lacking either CD4^+^ or CD8^+^ T-cells (10, 11).

In Chagas disease, subjects are considered infected when they test positive by at least two out of three serological tests (12). A significant proportion of subjects screened for *T. cruzi* infection have what is termed “discordant serology,” i.e., a positive result in only one out of three conventional serological tests. Additionally, a substantial number of individuals who live in regions of transmission are negative in the three serological tests but have measurable T-cell responses to *T. cruzi* (13). The existence of both of these subject groups strongly suggests that exposure to *T. cruzi* infection either sometime results in the induction of poor antibody responses or that a proportion of individuals exposed to *T. cruzi* actually resolve the infection. The latter hypothesis is further supported by the existence of subjects who spontaneously convert from seropositive (SP) to seronegative (14–19) and by the profile of drug-treated subjects who become seronegative but maintain memory T-cell responses (20, 21).

In this study, we measured the frequencies and quality of *T. cruzi*-specific CD4^+^ T-cells, as well as the levels of *T. cruzi*-specific antibodies using a multiplex assay format, in subjects with discordant findings by conventional serological tests but epidemiological evidence indicative of *T. cruzi* infection. We demonstrate that serodiscordant (SD) subjects exhibited a more robust *T. cruzi*-specific T-cell response compared with SP subjects, with the simultaneous production of interferon-γ (IFN-γ) and interleukin (IL)-2 in response to *T. cruzi* antigens and a resting phenotype. Low levels of antibodies specific for *T. cruzi*-derived recombinant proteins were also found in SD subjects, supporting the presence of a differential parasite-specific memory response in these individuals that is consistent with parasitological cure.

## Materials and Methods

### Selection of Study Population

Subjects with discordant or negative serology for *T. cruzi* infection but epidemiological findings for Chagas disease were recruited at the Chagas disease Unit of Hospital Interzonal de Agudos Eva Perón in Buenos Aires, Argentina. The protocol was approved by the IRB of the Hospital Interzonal de Agudos Eva Peron (Protocol No. 40/14 and 14-0004). Signed informed consent was obtained from all individuals prior to inclusion in the study following the guidelines of the United States Department of Health and Human Services. *T. cruzi* infection was determined by indirect immunofluorescence assay, hemagglutination, and ELISA techniques (12), by using an in-house epimastigote whole homogenate antigen, at the Instituto Nacional de Parasitología Dr. Mario Fatala Chabén, Argentina. Patients positive in at least two of these tests were considered to be infected, while subjects with positive results in one out of three tests, repeated at least twice, were considered as having discordant serology. Physical evaluation, electrocardiography and echocardiography were performed in all subjects included in the study. Twenty-one subjects with discordant serology, and 40 SP individuals, all born in areas endemic for *T. cruzi* infection (i.e., five subjects in Paraguay, and the remaining subjects in different endemic provinces of Argentina), but living in Buenos Aires by the time of the study were recruited for analysis. These individuals did not display clinical signs of *Leishmania* infection, which is endemic in some provinces of Argentina, and the amnanesis questionnaire did not revealed that SD subjects had been infected with *Leishmania*. Twelve subjects born and raised in Buenos Aires where *T. cruzi* infection is not endemic were recruited as uninfected control subjects not-exposed to *T. cruzi* (Table 1). Seven patients with Tegumentary Leishmaniasis infection or *leishmaniasis-T. cruzi* coinfection born and living in Salta, an argentinean province endemic for leishmaniasis, were also included as controls. Tegumentary Leishmaniasis is the prevalent type of *Leishmania* disease in the provinces in which SD and SP subjects for *T. cruzi* infection were born. Individuals with hypertension, ischemic heart disease, cancer, HIV infection, syphilis, diabetes, arthritis, or serious allergies were excluded from this study.

**Table 1:** Characteristics of study population.

Study group	*N*	Electrocardiographic findings^a^	Years of residence in non-endemic areas, median (range)	Age range^b^ (median), years	Gender	
					Male	Female
Serodiscordant	21^c,d^	2^e^	28 (11–47)	29–62 (43)	12	9
Seropositive	40^c^	0	29 (11–53)	30–63 (47)	15	25
Seronegative	12^f^	0	46 (38–61)	38–61 (46)	2	10

*^a^Number of subjects presenting electrocardiographic alterations*.

*^b^Age at entry of the study*.

*^c^All subjects were born and had lived for 16 years in average in areas endemic for T. cruzi infection but were living in Buenos Aires city by the time of the study*.

*^d^Seventeen subjects were born in provinces endemic for Leishmania sp*.

*^e^One subject with left anterior fascicular block; one subject with right bundle branch block plus left anterior fascicular block and grade II premature ventricular contractions according to Lown grading system (22)*.

*^f^All subjects were born and had lived in areas non-endemic for T. cruzi infection*.

### Collection of Peripheral Blood Mononuclear Cells (PBMC) and Serum Specimens

Approximately 50 mL of blood was drawn by venipuncture into heparinized tubes (Vacutainer; BD Biosciences). PBMC were isolated by density gradient centrifugation on Ficoll-Hypaque (Amersham) and stored in liquid nitrogen at 1.5 × 10^7^ million PBMC/mL in new-born bovine serum containing 10% DMSO. On the day of the assay, PBMC were thawed and washed in RPMI media containing 10% new-born bovine serum, 100 units/mL penicillin, 0.1 mg/mL streptomycin, 2 mM l-glutamine, and 10 mM Hepes buffer. Viability of cells was checked by trypan blue staining with a viability range of 80–90%. Another 10 mL of blood was allowed to coagulate at room temperature and centrifuged at 1,000 *g* for 15 min for serum separation. Due to sample availability the assays were not run for all the samples.

### *Trypanosoma cruzi* and *Leishmania* Antigens

Protein lysate from *T. cruzi* amastigotes derived from the Brazil strain was obtained by four freeze/thaw cycles followed by sonication, as previously reported (23). Recombinant His-tagged *T. cruzi*-derived proteins were expressed in *Escherichia coli* and purified under denaturing conditions using TALON Metal Affinity Resins, as previously described (24), including glycosomal phosphoenolpyruvatecarboxykinase (ATP) (TcCLB.508441.20) (KN104), putative 60S acidic ribosomal protein P0 (TcCLB.508355.250) (KN107), paraflagellar rod protein 2 (TcCLB.511215.119) (KN117), flagellar calcium-binding 24 kDa protein (TcCLB.507491.151) (KN122), putative surface protein TolT (TcCLB.510433.20) (ANOF), and conserved hypothetical protein (TcCLB.506635.130) (ANOH). *Leishmania* antigen from Montenegro skin test was kindly provided by the Instituto Nacional de Parasitología Dr. Mario Fatala Chabén, Argentina. A *Leishmania* lysate comprised by a mix of promastigotes derived from the predominant spp. found in the Northwest of Argentina, *Leishmania braziliensis* and *Leishmania amazonensis* was obtained as a second source of *Leishmania* antigen. Promastigotes of *L*. *braziliensis* and *L. amazonensis* were separately processed. Briefly, parasites were washed by centrifugation with phosphate-buffered saline at 4,500 rpm for 10 min. Deionized sterile water was added and five freeze/thaw cycles were performed followed by centrifugation at 4,500 rpm. Supernatant was stored at −80°C until use. Protein content was measured by Bradford method with a final protein concentration of 0.8 mg/mL.

### Interferon-γ and IL-2 Enzyme-Linked Immunosorbent Spot (ELISPOT) Assays

The number of *T. cruzi* antigen-responsive IFN-γ- and IL-2-secreting T-cells was determined by *ex vivo* ELISPOT assays, using a commercial kit (ELISPOT Human IFN-γ or IL-2 ELISPOT Set; BD Biosciences, USA), as described elsewhere (23, 25). PBMC were stimulated with 10 µg/mL of a *T. cruzi*-derived lysate preparation, 10 µg/mL *T. cruzi*-derived recombinant proteins, 10 µg/mL Montenegro *Leishmania* antigen, 20 ng/mL Phorbol 12-myristate 13-acetate (Sigma) plus 500 ng/mL ionomycin (Sigma), or media alone for 16–20 hr at 37°C and 5% CO_2_. Spot forming cells (SFCs) were automatically enumerated using ImmunoSpot analyzer (CTL). The mean number of spots in triplicate wells was obtained for each condition, and the number of specific IFN-γ and IL-2-secreting T-cells was calculated by subtracting the value of the wells containing media alone from the antigen-stimulated spot count. Responses were considered positive if (1) a minimum of 10 SFC/4 × 10^5^ PBMC were present per well and (2) this number was at least twice the value of wells with media alone (26).

### Intracellular Cytokine Staining for Polyfunctional T cells

The 4 × 10^6^ PBMC/well were stimulated with 15 µg/mL *T. cruzi* amastigote lysate or media alone plus 1 µg/mL CD28/CD49d (BD Biosciences) at 37°C in a CO_2_ incubator for 16–20 h. Stimulation with Staphylococcal enterotoxin B (1 µg/mL; Sigma Aldrich) served as a positive control. Brefeldin A was added for the last 5 h of incubation and then the cells were harvested and stained with antihuman CD4 allophycocyanin (APC-Cy7, clone RPA-T4) and antihuman CD3 Pacific Blue (clone UCHT1), both from BD Biosciences. After washing, cells were fixed and permeabilized with Cytofix/Cytoperm solution (BD Biosciences) and incubated with anti-human CD154-fluoresceinisothiocyanate (FITC, clone TRAP1), IFN-γ phyco-erythrin (PE, clone 4S.B3), tumor necrosis factor (TNF)-α allophycocyanin (APC, clone 6401.1111), macrophage inflammatory protein (MIP)-1β peridinin chlorophyll (PerCP Cy5, clone D21-1351), and IL-2 (PECy7, MQ1-17H12) (BD Biosciences). Approximately 600,000 events were acquired per sample in a HyperCyAn flow cytometer (Beckman Coulter, Brea, CA, USA). Analysis was performed with FlowJo software (Tree Star Inc., Ashland, OR, USA). Lymphocytes were gated based on forward scattering (FSC) and side scattering (SSC), and CD3^+^ cells were then analyzed for CD4 vs. each marker. Cytokine coexpression profiles were determined using the Boolean gating function of FlowJo software (see Figure S1 in Supplementary Material). A total of 31 different T-cell populations were obtained from the combination of the five different T-cell functions analyzed. Background responses detected in negative control samples with media alone were subtracted from those detected in *T. cruzi*-stimulated samples for every specific functional combination. Negative cutoff values for each specific functional combination were calculated as the average *T. cruzi*-specific T-cell response plus three standard deviations in two seronegative subjects. T-cell responses for each functional combination were considered positive when the T-cell response was higher than the cutoff value and at least twice the response with media alone; at least five events were present for single responses or at least three events for polyfunctional responses (27). For analysis only individuals with positive T-cell responses to *T. cruzi* antigens for at least one coexpression profile were included when calculating the proportion of each cytokine-producing subset contributing to the total *T. cruzi*-specific response. The mean fluorescence intensity (MFI) of each cytokine for different cytokine coexpression subsets was analyzed to determine the amount of cytokines produced in response to *T. cruzi*.

### Cell-Surface Staining for Phenotypic Markers

The 1 × 10^6^ PBMC were stained with a panel of antihuman-CD4 (PerCP) or anti-CD8 (PerCP), anti-CD45RA (FITC), anti-CD127 Alexa Fluor 647 and anti-CD132 (PE); or a panel of anti-CD4 (PerCP) or anti-CD8 (FITC) and anti-HLADR (PE) for 30 min on ice. Data were acquired on a FACS Calibur flow cytometer (Becton Dickinson, USA) and analyzed with FlowJo software (Tree Star, San Carlos, CA, USA).

### Antibody Testing by Multiplex Assay

Serum specimens were screened for antibodies reactive to a panel of 11–14 recombinant *T. cruzi* proteins in a Luminex-based format, as previously described (20, 24). The ratio of the specific MFI for each antigen to the MFI of the negative control protein was calculated for each serum. Values above de mean plus four standard deviations of a minimum of 16 true negative sera run in the same assay, and individually determined for each antigen were considered a positive test.

### Statistics

Demographic and clinical characteristics of *T. cruzi*-infected subjects included in this study were summarized using range and median. The normality of data was evaluated by the Shapiro–Wilk test. Differences among groups were evaluated by ANOVA followed by a Bonferroni test for multiple comparisons or by a test for linear trend. Comparisons of the frequencies of responders in the different categories of ELISPOT responses between groups were evaluated by use of the chi^2^ test or Fisher’s exact test, as appropriate. The Mann–Whitney *U* test or Student’s *t*-test was applied to compare the number of spot counts, the number of functions and the MFI of each cytokine between SD and SP individuals. Differences were considered statistically significant at *P* < 0.05.

## Results

### Clinical and Serological Characteristics of Subjects with Positive, Discordant, or Negative Conventional Serological Tests for *T. cruzi* Infection at Baseline

Serodiscordant subjects had positive results for *T. cruzi*-specific antibodies by immunofluorescence (i.e., 15 out 20 SD subjects) or hemagglutination (i.e., 6 out of 21 SD subjects) but negative findings by ELISA (Table 2). Two SD subjects showed some degree of electrocardiographic alterations but had normal left ventricular diameters and normal global systolic function, indicating absence of heart failure (Table 1). The remaining subjects presented no clinical or electrocardiographic abnormalities (Table 1).

**Table 2:** Humoral and T-cell responses to *T. cruzi* antigens in subjects with discordant conventional serology.

Subject ID	Months of follow-up	Conventional serologic tests			No. spots/4 × 10^5^ PBMC		Multiplex assay^d^
		ELISA^a^	IHA^b^	IIF^b^	IFN-γ^c^	IL-2^c^	

		OD	Titer	Titer			
PP47	0	0.126	NR	32	48^e^	12^e^	0/11
	24	0.168	16	NR	29^e^	12^e^	0/11
	36	0.165	**32**	**64**	10^e^	10^e^	0/11
	60	0.127	16	32	ND	ND	1/11
	72	0.120	16	64	ND	ND	2/12
	84	0.140	NR	NR	ND	ND	ND

PP48	0	0.086	16	32	23^e^	17^e^	1/11
	12	0.096	NR	NR	318^e^	ND	1/11
	24	0.031	16	NR	ND	ND	1/11
	36	0.043	16	NR	ND	ND	2/11

PP106	0	0.069	NR	64	14^e^	ND	1/11
	12	0.084	**64**	**128**	33^e^	10^e^	ND
	24	0.099	NR	128	70^e^	25^e^	3/11
	36	0.048	64	NR	19^e^	13^e^	2/11
	48	0.069	64	NR	26^e^	12^e^	1/11

PP127	0	0.085	NR	64	115^e^	1	3/14
	12	0.081	NR	NR	30^e^	ND	2/14
	36	0.089	**32**	**128**	15^e^	0	2/14
	60	0.113	NR	NR	10^e^	0	4/14

PP177	0	0.150	16	128	40^e^	ND	2/11
	12	0.145	16	128	42^e^	ND	2/11
	24	0.109	NR	64	37^e^	10^e^	1/11
	36	0.141	NR	32	142^e^	30^e^	1/11
	48	0.075	NR	NR	33^e^	5	0/11
	60	0.137	NR	128	24^e^	10^e^	0/11
	84	0.156	NR	NR	40^e^	13^e^	1/11

*Time points with two positive conventional serological tests out of three performed are indicated in bold*.

*^a^Threshold of reactivity for ELISA, 0.200*.

*^b^Threshold of reactivity for indirect hemagglutination (IHA) and indirect immunofluorescence (IIF), ≥1/32*.

*^c^Spot counts with media alone were subtracted*.

*^d^Number of reactive proteins out of total proteins assessed*.

^e^Indicates positive response as described in Section “Materials and Methods.”

*NR, no reactive; ND, not done*.

### Presence of *T. cruzi*-Specific T-Cell Responses in Subjects with Discordant Serology for Chagas Disease

**Figure 1:**
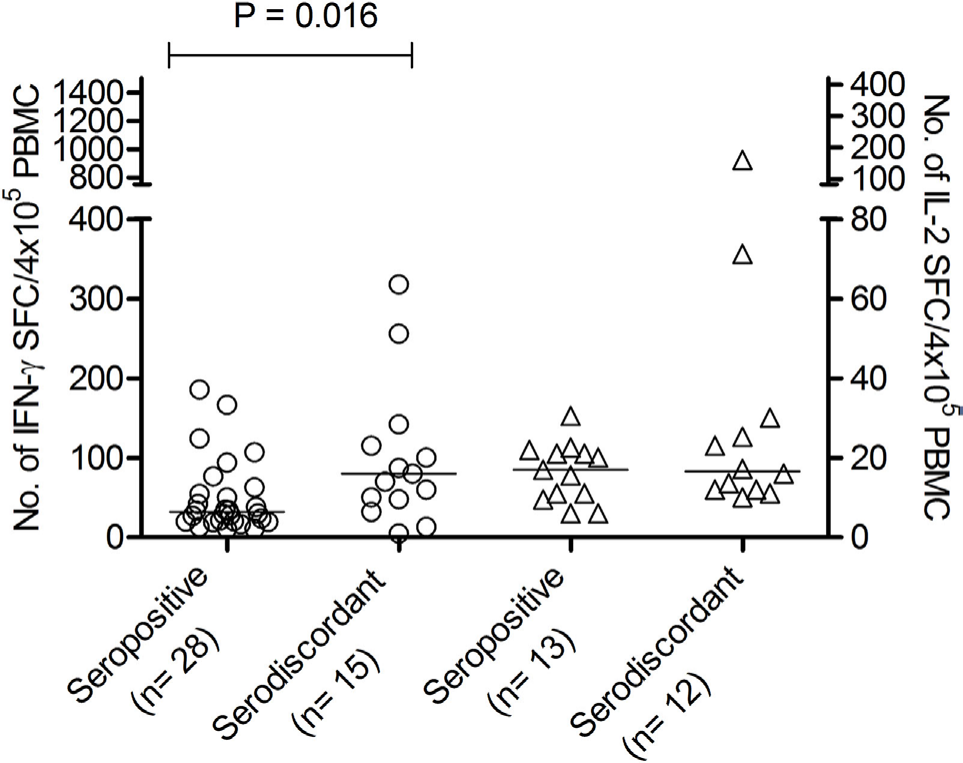
Number of interferon (IFN)-γ and interleukin (IL)-2-spot forming cells (SFCs) in response to *Trypanosoma cruzi* antigens in serodiscordant and seropositive subjects for *T. cruzi* infection. Peripheral blood mononuclear cell (PBMC) were stimulated with a *T. cruzi* lysate from the Brazil strain or media alone for 16–20 h. Each symbol represents the mean spot number of triplicate wells for each subject with positive IFN-γ (circles, left *Y* axis) or IL-2 (triangles, right *Y* axis) enzyme-linked immunosorbent spot (ELISPOT) responses, as defined in material and methods. Spot counts with media alone were subtracted. horizontal lines depicting median values are shown. Significant differences between subject groups were determined by Mann–Whitney *U* test.

We have previously shown that adult subjects with chronic *T. cruzi* infection have IFN-γ-producing T-cells responsive to *T. cruzi* antigens whereas IL-2 secreting cells are rare in Chagas disease patients. In contrast, uninfected non-exposed subjects have no T-cell responses specific for *T. cruzi* (23, 25, 28, 29). Herein, *T. cruzi*-specific T-cell responses measured by direct *ex vivo* cytokine production in response to a *T. cruzi* lysate were evaluated in 19 subjects with discordant findings in conventional serological tests. Most subjects with discordant serology had T-cells that showed simultaneous secretion of both IFN-γ and IL-2 in response to *T. cruzi* (Tables 2 and 3), contrasting with the low prevalence of IL-2-producing T-cells in SP individuals (Table 3). The number of IFN-γ spot counts among responder subjects by ELISPOT was significantly higher in SD subjects than in SP subjects (Figure 1). Although not statistically significant, a trend to higher IL-2 spot counts was also observed in SD subjects compared with SP individuals.

**Table 3:** Cytokine-secreting T cells in response to a *T. cruzi* lysate in subjects with discordant, positive, or negative findings by conventional serological techniques living in non-endemic areas.

ELISPOT responses to *T. cruzi* lysate	No. of positive responders/total no. of subjects evaluated (%)		
	Serodiscordant^a^	Seropositive^a^	Uninfected^b^
IFN-γ^+^IL-2^+^	12/19 (63.1)^c^	13/40 (32.5)^d^	0/12 (0)
IFN-γ^+^IL-2^−^	3/19 (15.7)	15/40 (37.5)^e^	0/12 (0)
IFN-γ^−^IL-2^−^	4/19 (21.0)	12/40 (30)^f^	0/12 (0)

*^a^Subjects were born and had lived in areas endemic for T. cruzi infection for 16 years in average, but were living in Buenos Aires city by the time of the study*.

*^b^Subjects were born and had constantly lived in areas non-endemic for T. cruzi-infection*.

*^c^*P* = 0.04 vs. percentage of responders in the seropositive group; *P* = 0.0004 vs. percentage of responders in the uninfected group; *P* = 0.02 vs. IFN-γ^+^IL-2^−^ responders in the serodiscordant group; *P* = 0.0176 vs. IFN-γ^−^IL-2^−^ non-responders in the serodiscordant group (Fisher’s exact test)*.

*^d^*P* = 0.0243 vs. the uninfected group (Fisher’s exact test)*.

*^e^*P* = 0.0119 vs. the uninfected group (Fisher’s exact test)*.

*^f^*P* = 0.047 vs. the uninfected (Fisher’s exact test)*.

**Figure 2:**
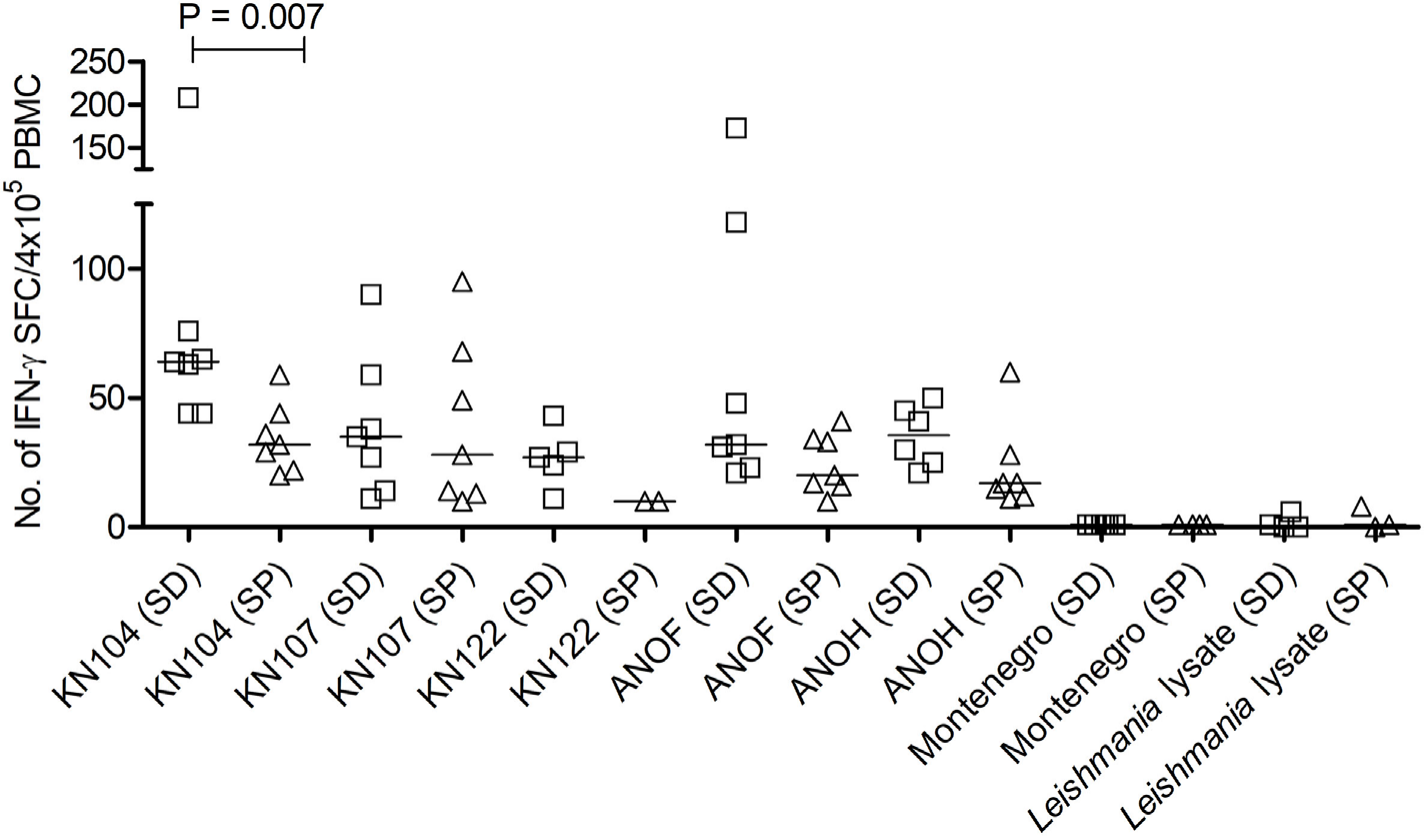
Number of interferon (IFN)-γ–secreting cells in response to *Trypanosoma cruzi*-derived recombinant proteins in serodiscordant and seropositive subjects for *T. cruzi* infection. Peripheral blood mononuclear cell (PBMC) were seeded at 4 × 10^5^ cells/well and stimulated with *T. cruzi*-derived recombinant proteins and Montenegro *Leishmania* antigen or media alone, for 16–20 h. Each symbol represents the mean spot number of triplicate wells for each serodiscordant (squares) or seropositive (triangles) with positive IFN-γ enzyme-linked immunosorbent spot (ELISPOT) responses to the indicated *T. cruzi*-derived recombinant protein, as defined in material and methods. Spot counts with media alone were subtracted. Horizontal lines depicting median values are shown. Responses to Montenegro *Leishmania* antigen or in house *Leishmania* lysate were measured as negative controls. Significant differences between subject groups were determined by Mann–Whitney *U* test.

To confirm the specificity of T-cell responses in SD subjects, the frequency of IFN-γ-producing T-cells in response to a set of *T. cruzi*-derived recombinant proteins included in a multiplex assay developed by our group was also evaluated. These proteins were selected for being high-abundant in the amastigote and trypomastigote stages or being unique for *T. cruzi* (24). Eight out of nine (88.9%) SD subjects showed positive responses to 3–5 proteins assessed, while IFN-γ-producing T-cells were present in 11 out of 15 (73.3%) SP subjects in response to 1–5 proteins. Positive responses to *T. cruzi*-derived recombinant proteins were not observed in the 10 seronegative subjects not exposed to *T. cruzi* evaluated (data not shown). SD subjects had higher levels of IFN-γ producing T-cells responsive to the KN 104 recombinant protein compared with SP individuals (Figure 2). In addition, higher frequency of IFN-γ-producing T-cells specific for KN122, ANOF, and ANOH was observed in SD subjects compared with SP subjects, although the differences were not statistically significant (Figure 2). Of note, seven SD subjects showed responses to ANOF, a protein unique to *T. cruzi*. T cells in SD and SP subjects did not recognize *Leishmania* antigens (Figure 2). In addition, T cells in patients with Tegumentary leishmaniasis did not recognize *T. cruzi* antigens, while patients with *Leishmania-T. cruzi* coinfection presented positive T-cell responses to the *T. cruzi* lysate further corroborating that T-cell responses in SD subjects are specific for *T. cruzi* (Table 4). Thus, a subset of SD subjects exhibit robust T cells responsive to *T. cruzi* suggesting prior exposure to the parasite.

**Table 4:** IFN-γ and IL-2-secreting T-cells in response to a *T. cruzi* lysate in patients with Tegumentary leishmaniasis.

Subject ID	Parasite infection	No. spots/4 × 10^5^ PBMC^a^	
		IFN-γ	IL-2
1	*Leishmania*	0 (−)	1 (−)
2	*Leishmania*	4 (−)	1 (−)
3	*Leishmania*	1 (−)	0 (−)
4	*Leishmania-T. cruzi*^b^	116 (+)	1 (−)
5	*Leishmania-T. cruzi*^b^	46 (+)	0 (−)
6	*Leishmania-T. cruzi*^b^	49 (+)	4.5 (−)
7	*Leishmania-T. cruzi*^b^	289 (+)	34 (+)

*^a^Spot counts with media alone were subtracted. Positive (+) and negative (−) responses as defined in material and methods are indicated*.

*^b^Subjects with confirmed diagnosis for Leishmania infection bearing positive serology for T. cruzi infection by conventional serological tests*.

### Polyfunctional T-Cell Responses in SD Subjects

**Figure 3:**
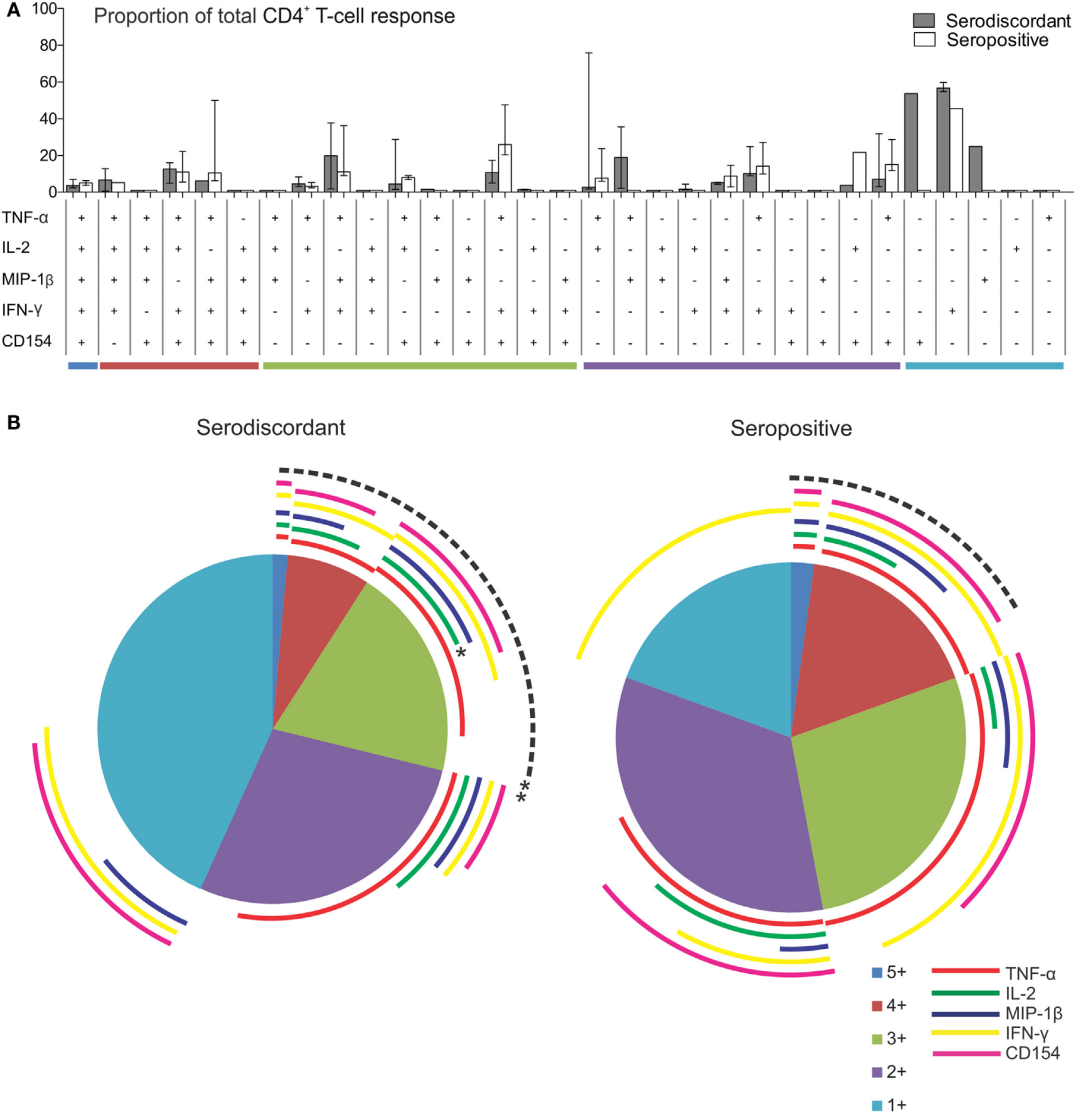
Functionality of *Trypanosoma cruzi*-specific CD4^+^ T cells in serodiscordant and seropositive subjects for *T. cruzi* infection. Peripheral blood mononuclear cells (PBMC) were stimulated with a *T. cruzi* lysate preparation from the Brazil strain and analyzed by flow cytometry for intracellular expression of interferon (IFN)-γ, interleukin (IL)-2, tumor necrosis factor (TNF)-α; macrophage inflammatory protein (MIP)-1β and CD154 in CD4^+^ T-cells. Lymphocytes were gated based on forward scattering (FSC) and side scattering (SSC), and CD3^+^ cells were then analyzed for CD4 vs. each marker. Cytokine coexpression profiles with one (1+), two (2+), three (3+), four (4+), and five (5+) functions were determined in serodiscordant and seropositive subjects, using the Boolean gating function of FlowJo software. **(A)** The contribution of each cytokine-producing subset to the total *T. cruzi*-specific CD4 T-cell response was assessed in donors with positive responses in the intracellular staining assay as indicated in material and methods (i.e., nine serodiscordant subjects and eight seropositive subjects). Then, the average for each combination was calculated. Bars indicate the percentage of the total response contributed by CD4^+^ T-cells with a given functional response. Data are represented as median percentages with interquartile range. Responses for all 31 possible subsets are shown for serodiscordant and seropositive subjects. **(B)** The data are summarized by the pie charts, in which each slice of the pie represents the fraction of the total response that consists of CD4^+^ T-cells positive for one to five functions (light blue, violet, green, red, and blue, respectively), matched to the horizontal bars in A. The arches indicate the proportion of CD4^+^ T-cells that express CD154 (pink), IFN-γ (yellow), MIP-1β (blue), IL-2 (green), or TNF-α (red) in each slice of the pie. **P = 0.006 compared with the fraction of IL-2 producing cells on the total polyfunctional response in seropositive subjects; *P = 0.02 compared with the fraction of IL-2-producing cells in the slice of the pie with three functions in seropositive subjects, by Student’s *t*-test.

To further assess the functional capacity of *T. cruzi*-specific T-cells in SD subjects in comparison to SP subjects, multiparameter flow cytometry was performed to measure five T-cell functions, IFN-γ, IL-2, TNF-α, MIP-1β, and CD154, independently and simultaneously in these groups. The analysis was focused on CD4^+^ T-cells since this is the main T-cell population induced by the amastigote lysate preparation (23, 25). We first analyzed the contribution of each subset to the total CD4^+^ T-cell response in subjects with positive responses by intracellular staining. Although no statistically significant, SD subjects displayed an increased proportion of monofunctional CD4^+^ T-cells compared to SP subjects (Figures 3A,B). *T. cruzi*-specific monofunctional CD4^+^ T-cell responses from SD subjects are predominantly comprised of IFN-γ, CD154 and MIP-1β-producing cells, whereas SP subjects had primarily IFN-γ-producing cells (Figures 3A,B).

**Figure 4:**
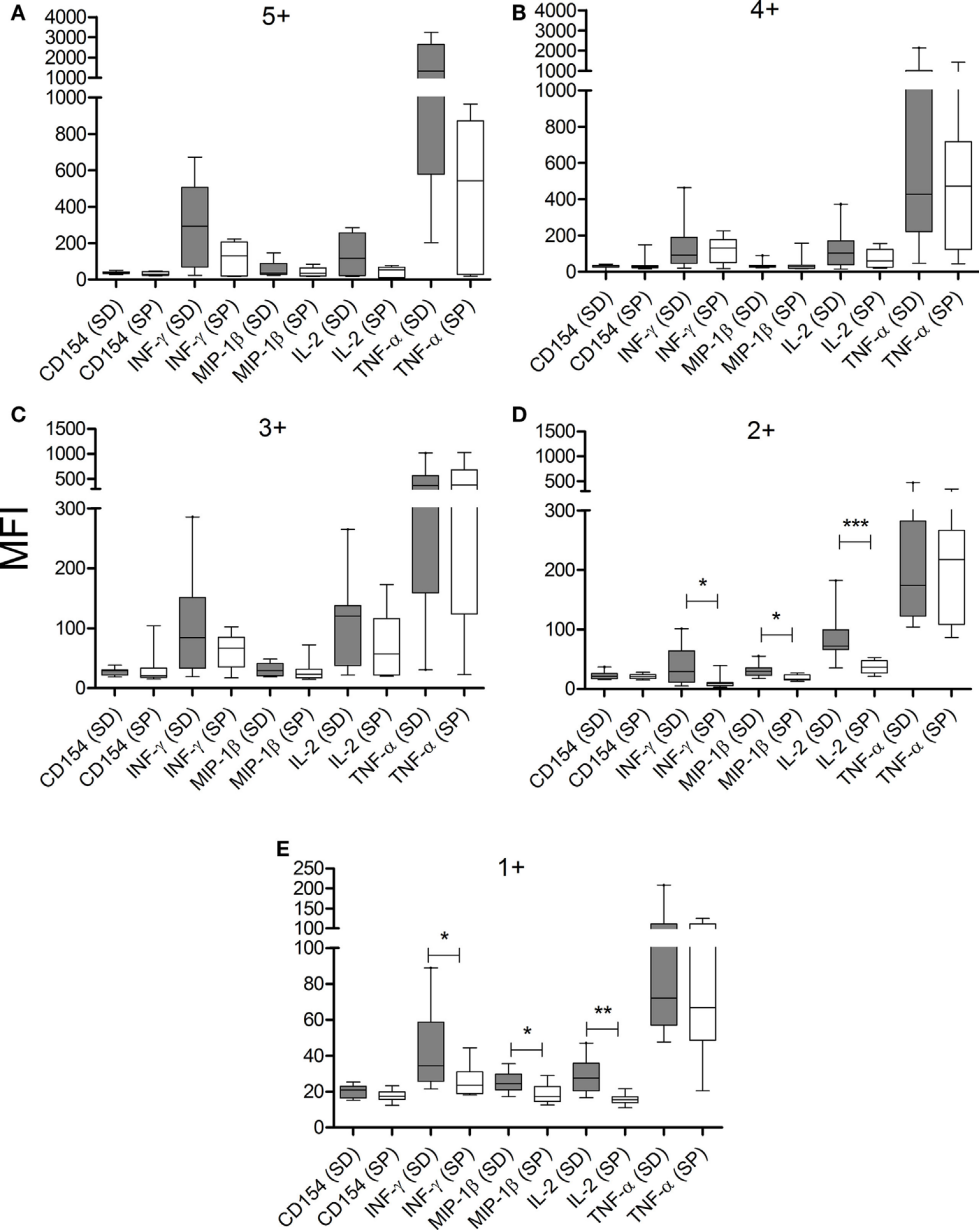
CD4^+^ T cells from serodiscordant subjects express higher amounts of cytokines than seropositive individuals in response to *T. cruzi* antigens. Peripheral blood mononuclear cells (PBMC) were stimulated with a *T. cruzi* lysate preparation from the Brazil strain and *T. cruzi*-specific CD4 T-cell responses were measured using an intracellular staining assay. The mean fluorescence intensity (MFI) of each cytokine for different cytokine coexpression subsets was analyzed. The median values and 10–90 percentile of MFI of each cytokine for CD4^+^ T cells with 5+ **(A)**, 4+ **(B)**, 3+ **(C)**, 2+ **(D)**, or 1+ **(E)** function were calculated. MFI of each cytokine was compared between serodiscordant and seropositive individuals by Mann–Whitney *U* test. (SD) Serodiscordant subjects; (SP) seropositive subjects. ***P < 0.001; **P < 0.01; *P < 0.05 by Mann–Whitney *U* test or Student’s *t*-test.

The composition of polyfunctional T cells in SD subjects was also different from that in SP individuals, with an enrichment in IL-2-producing T-cells (i.e., 52 and 21% of the total CD4^+^ T-cells producing cytokines consisted of IL-2-producing cells, respectively, black arch in Figures 3A,B). This is particularly noticeable in CD4^+^ T-cells exhibiting three functions (i.e., green arch in Figures 3A,B). Moreover, the magnitude of IL-2 production by CD4^+^ T-cells in SD subjects, as determined by the MFI, was higher than those in SP subjects (Figures 4A–E). SD subjects also showed a higher production of IFN-γ and MIP-1β among CD4^+^ T-cells expressing one (Figure 4E) or two (Figure 4D) functions compared with SP individuals.

### Resting Status of CD4^+^ and CD8^+^ T cells in SD Subjects

We have previously demonstrated that *T. cruzi*-infected subjects have decreased frequencies of T cells expressing the IL-7R components CD127^+^CD132^+^ and a reciprocal gain of CD127^-^CD132^+^ in CD8^+^ and CD4^+^ T-cells when compared with uninfected subjects, supporting a process of persistent antigen stimulation (30). *T. cruzi*-infected subjects also exhibit an increased number of CD4^+^ and CD8^+^ T-cells expressing the activation marker HLA-DR (31). To explore whether SD subjects had a T-cell profile more similar to actively infected or to uninfected subjects, the expression of molecules denoting chronic immune activation and antigen exposure was evaluated in these groups.

**Figure 5:**
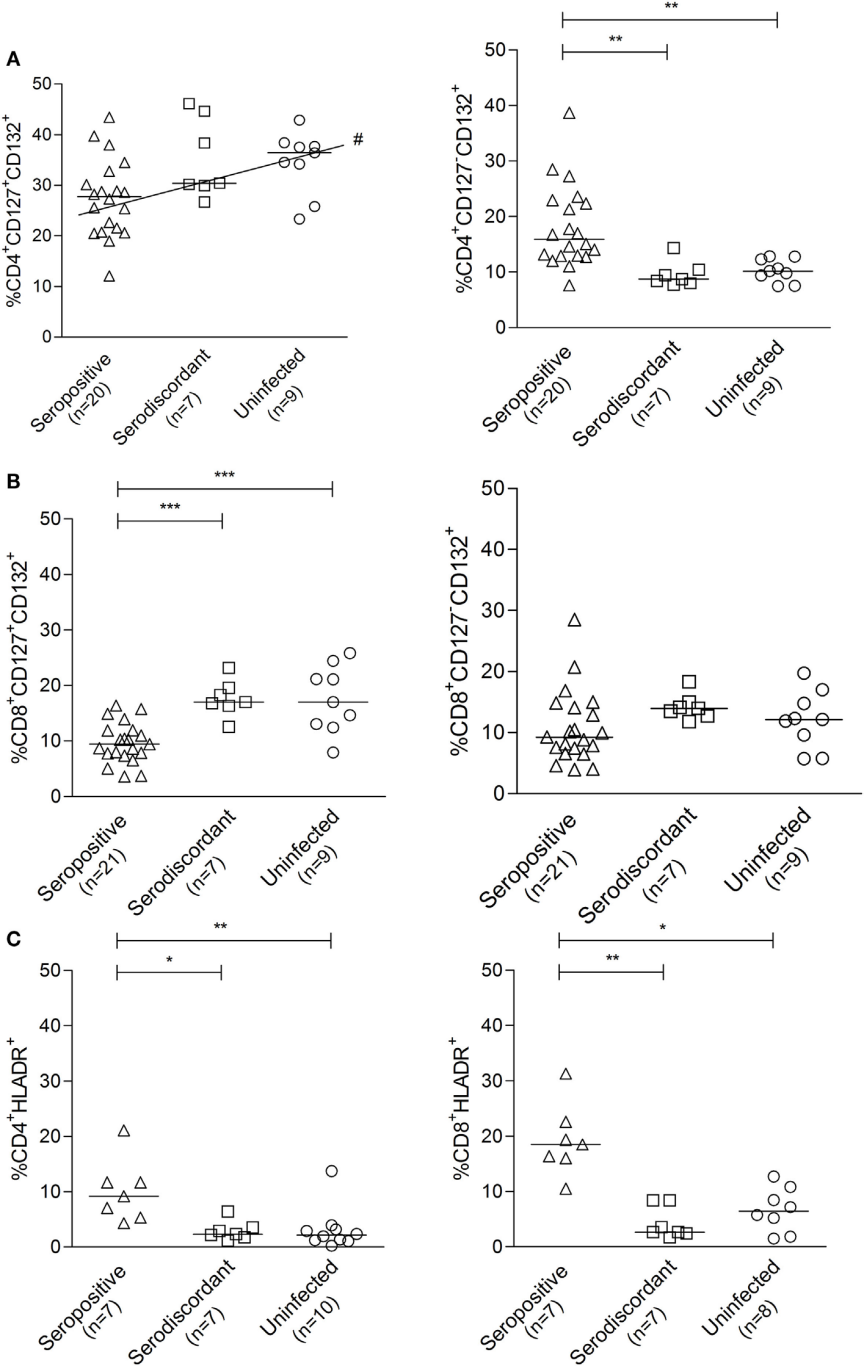
Cell surface expression of the interleukin (IL)-7 receptor components and HLA-DR in subjects with serodiscordant serology for *Trypanosoma cruzi* infection. Peripheral blood mononuclear cells (PBMC) were stained for CD8, CD4, CD127, CD132, and HLA-DR and analyzed by flow cytometry. Lymphocytes were gated by forward and side scattering. Each point represents the expression of CD127 and CD132 **(A,B)** or HLA-DR **(C)** on total CD4^+^ and CD8^+^ T cells for individual subjects. Median values are indicated by the horizontal lines. *P < 0.05; **P < 0.001; ***P < 0.0001, # positive trend *P* < 0.05 for CD4^+^CD127^+^CD132^+^ T cells. Differences among groups were evaluated by ANOVA or Kruskal–Wallis test followed by posttests.

The frequencies of CD127^+^ CD132^+^ and CD127^-^CD132^+^ cells in the CD4^+^ (Figure 5A) and CD8^+^ (Figure 5B) T-cell compartments in SD subjects were similar to those found in uninfected non-exposed subjects. The expression of HLA-DR in CD4^+^ and CD8^+^ T-cells in SD subjects was also comparable to that in uninfected controls and distinct from SP individuals (Figure 5C). These findings indicate that T cells in SD individuals have a resting phenotype different from that of SP individuals with active infection.

### Humoral Immune Responses Specific for *T. cruzi*-Derived Recombinant Proteins

**Figure 6:**
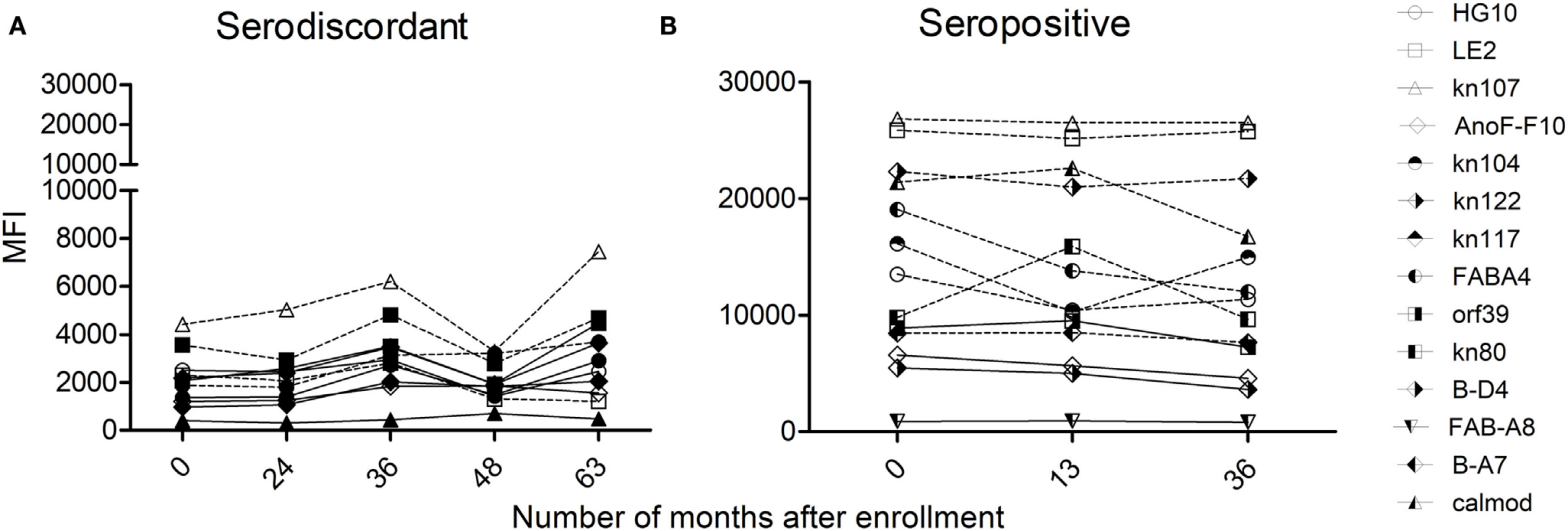
Monitoring of antibody responses specific for *Trypanosoma cruzi* by multiplex assays in serodiscordant or seropositive subjects for *T. cruzi* infection. Mean intensity fluorescence (MFI) of sera from serodiscordant **(A)** and seropositive **(B)** subjects to a panel of 11–14 recombinant *T. cruzi* proteins. Reactive proteins (i.e., MFI four standard deviations above that of a set of true negative sera, 24) are indicated with dotted lines. Plots show representative data for single subjects.

Humoral immune responses were also assessed by multiplex assay (24), which allowed the detection of antibodies specific for a set of *T. cruzi*-derived recombinant proteins. The majority of SD subjects (i.e., 14/19 subjects assayed) had low antibody responses specific for *T. cruzi*-derived recombinant proteins compared with SP subjects, in terms of both the number of proteins recognized and the strength of reaction (Table 2, Figures 6A,B) (24). SD subjects had positive reactivity to 1.5 proteins in average, compared with more than four proteins in SP subjects detected herein (Figure 6B) and in former studies (24, 31, 32).

### Monitoring of *T. cruzi*-Responsive T cells and Humoral Responses in Subjects with Discordant Serology

T-cell and antibody responses against *T. cruzi* antigens in subjects with discordant serology for *T. cruzi* infection were sustained over time, as observed by the monitoring of five subjects in which these responses were evaluated for 3–8 years (Table 2). However, discordant serological findings by conventional serology might fluctuate between completely negative findings (i.e., three negative tests out of three performed) to positive testing by immunofluorescence or hemagglutination but not by ELISA assay (i.e., two positive tests out of three performed indicated in bold, Table 2). Likewise, *T. cruzi*-specific antibody levels also oscillated in a low range of responses by the multiplex technique (Table 2; Figure 6A). SD subjects did not show electrocardiographic changes during the follow-up period.

## Discussion

Spontaneous cure of *T. cruzi* infection is considered a rare event. However, cases of consistently negative conventional serological tests along with the lack of positive parasitological tests in subjects with evident acute infection who have never received etiological treatment have been reported (14–16). Seroreversion has also been observed in long-term outcomes of chronically *T. cruzi*-infected subjects without treatment intervention (17, 18). The observation of subjects with inconclusive serological findings, including those with one positive conventional test out of three performed, raises the question of whether these subjects have cleared the infection.

This study provides the first documentation showing T-cell immunity to *T. cruzi* in subjects with discordant serologic findings for Chagas disease. However, the functional capacity of T-cells responsive to *T. cruzi* antigens is different between SD and SP subjects. A higher prevalence of IFN-γ^+^IL-2^+^ responders as well as a higher magnitude of these responses was found in SD individuals than in SP subjects. These findings are at odds with the low frequency of IL-2-producing T-cells in documented SP adult subjects (e.g., positive on all serological tests) observed herein, and in our previous studies (25, 33).

Multiparameter flow cytometry confirmed that polyfunctional *T. cruzi*-responsive T cells in SD subjects are enriched in highly competent IL-2-producing (34–36) *T. cruzi*-specific CD4^+^ T cells, consistent with exposure to *T. cruzi* but the absence of a long-term active infection. During chronic infections a hierarchical loss of different T-cell functions has been observed with the production of IL-2 being the first function compromised followed by the ability to make TNF-α, while IFN-γ production was most resistant to functional exhaustion upon persistent antigen stimulation (37). Therefore, the emergence of IL-2 producing T cells might occur after cessation of continuous antigen stimulation. Furthermore, polyfunctional T cells in *T. cruzi*-SD subjects also display stronger IFN-γ and MIP-1β production compared with SP subjects. In a recent study, we have shown that successful therapy with benznidazole resulted in a change of the functional profile of parasite-specific T-cells along with declining serology (21). Although IL-2 had not been assessed, the frequency of CD4^+^IFN-γ^+^ and CD4^+^CD154^+^ T cells increased after treatment compared with the levels prior to treatment (21).

The results we report here for *T. cruzi* infection are similar to those reported in other infections. For example, subjects able to spontaneously resolve hepatitis C virus (HCV) infection have demonstrated stronger IL-2 secretion in response to HCV antigens than persistently infected subjects (38). Polyfunctional T cells have also been observed in subjects potentially exposed to human immunodeficiency virus or HCV but seronegative for these infections (39, 40) and in populations naturally exposed to *Plasmodium falciparum* (41). Thus, one possible explanation for the presence of *T. cruzi*-specific T cells in subjects with discordant conventional serology is that they had been transiently infected with *T. cruzi* but cleared the infection with the maintenance of cellular and humoral immune memory.

Another explanation of the inconclusive serology in some subjects is the slow loss of anti-*T. cruzi* antibodies over time after prior clearance of the infection with the preservation of parasite-specific memory T cells. In a 10-year follow-up study of subjects with positive serology for *T. cruzi* infection, Sabino et al. showed that the levels of *T. cruzi*-specific antibodies declined over time in subjects with persistent negative PCR results (18). The presence of low levels of *T. cruzi* antibodies in SD subjects was confirmed by the low number of proteins reactive by the multiplex technique and the fact that these subjects only tested positive by the high sensitive immunofluorescence and hemagglutination techniques. The long-term follow-up of SD subjects showed that humoral and cellular responses might fluctuate but are sustained over time.

A third possibility to explain the inconsistent antibody levels in discordant subjects is that the antibodies are induced by another infection but are mildly cross-reactive with *T. cruzi*. Since several provinces of Argentina are endemic for both *T. cruzi* and *Leishmania* infections (12) and the presence of B-cell epitopes shared between *Leishmania* and *T. cruzi* has been reported (42–46), T-cell responses to two different sources of *Leishmania* antigens were evaluated in SD subjects. These experiments showed no evidence of IFN-γ-responsive T-cells specific for *Leishmania*. In addition, SD individuals showed humoral and T-cell responses to some recombinant proteins that are unique to *T. cruzi* and no present in *Leishmania*. Further, several SD subjects were born in provinces non-endemic for *Leishmania*. Of note, T cells in patients with Tegumentary Leishmaniasis did not recognize the *T. cruzi* lysate, further supporting the specific recognition of *T. cruzi* antigens in subjects exposed to *T. cruzi*.

Subjects chronically infected with *T. cruzi* not only display a functional impairment of T-cells but also increased frequency of terminally differentiated and activated T-cells (23, 25, 28, 29), indicating continuous antigenic stimulation presumably as a result of persistent infection. T-cells in SD individuals have a resting status as indicated by the lower levels of T cells expressing the HLA-DR activation marker or showing downregulation of the IL-7R compared with *T. cruzi*-infected subjects (30), consistent with a lack of recent antigen stimulation of T cells. Furthermore, SD subjects have no signs of heart failure or electrocardiographic changes over a followed period of up to eight years. Altogether, these data support that SD individuals might have been previously infected with *T. cruzi* and have apparently resolved that infection with the development of immunological memory.

## Ethics Statement

Signed informed consent was obtained from all individuals prior to inclusion in the study following the guidelines of the United States Department of Health and Human Services. The protocol was approved by the IRB of the Hospital Interzonal de Agudos Eva Peron (Protocol No. 40/14 and 14-0004).

## Author Contributions

All authors have participated to conception and design, analysis and interpretation of data, critically revision, or final approval of the version to be published.

## Conflict of Interest Statement

The authors declare that the research was conducted in the absence of any commercial or financial relationships that could be construed as a potential conflict of interest.

## Supplementary Material

Figure S1 Gate strategy for the analysis of cytokine coexpression profiles of CD4^+^ T cells following stimulation with *T. cruzi* lysate. Lymphocytes were gated based on forward (FSC) and side scattering (SSC), CD3^+^ population was then selected and CD4^+^ T-cells were analyzed for TNF-α, IL-2, MIP-1β, IFN-γ and CD154 expression. Cytokine coexpression profiles were determined using the Boolean gating function of Flow Jo software.
