# Supplementary figures and images for: The Significance of Discordant Serology in Chagas Disease: Enhanced T-Cell Immunity to Trypanosoma cruzi in Serodiscordant Subjects
Source: Front Immunol. 2017 Sep 15;8:1141. doi: 10.3389/fimmu.2017.01141 (PMC5605634; doi:10.3389/fimmu.2017.01141)

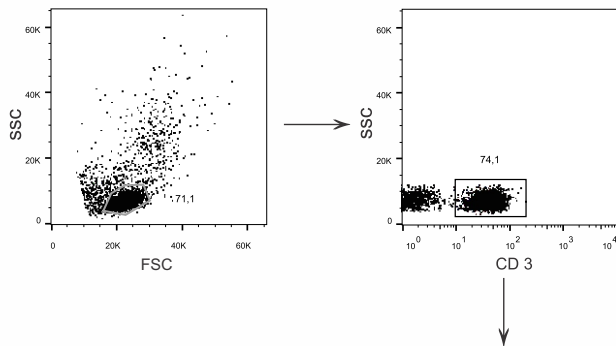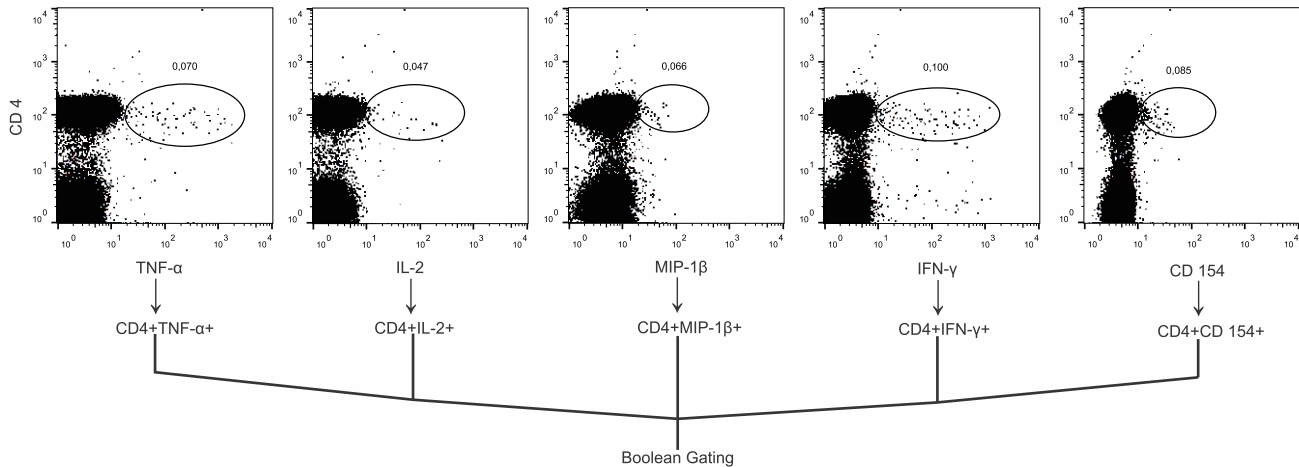

Boolean Gating

|               |   |   |   |   |   |   |   |   |   |   |   |   |   |   |   |   |   |   |   |   |   |   |   |   |   |   |   |
|---------------|---|---|---|---|---|---|---|---|---|---|---|---|---|---|---|---|---|---|---|---|---|---|---|---|---|---|---|
| TNF- $\alpha$ | + | + | + | + | + | - | + | + | + | - | + | + | - | + | - | - | + | + | - | - | - | + | - | - | - | - | + |
| IL-2          | + | + | + | + | - | + | + | + | - | + | + | - | + | - | + | - | + | - | + | + | - | - | - | - | - | - | + |
| MIP-1 $\beta$ | + | + | + | - | + | + | + | - | + | + | - | + | + | - | + | - | + | + | - | + | - | + | - | - | - | + | - |
| IFN- $\gamma$ | + | + | - | + | + | + | - | + | + | + | - | - | - | + | + | - | - | - | + | + | + | + | - | - | - | + | - |
| CD154         | + | - | + | + | + | + | - | - | - | - | + | + | + | + | + | + | - | - | - | - | - | + | + | + | + | + | - |

Supplement: Figure S1 — Gate strategy for the analysis of cytokine coexpression profiles of CD4+ T cells following stimulation with T. cruzi lysate. Lymphocytes were gated based on forward (FSC) and side scattering (SSC), CD3+ population was then selected and CD4+ T-cells were analyzed for TNF-α, IL-2, MIP-1β, IFN-γ and CD154 expression. Cytokine coexpression profiles were determined using the Boolean gating function of Flow Jo software. [file image_1.pdf]
